# “No”

**Published:** 1871-03

**Authors:** 


Not long since an article went the rounds of the newspapers showing how a man became immensely wealthy by having learned in early life when to say " No." If an old friend asked him to lend him some money, he uniformly replied " No." If a widow desired him to go her security for a load of wood in midwinter to prevent her and her fatherless little ones from perishing with cold, he said " No." If a friendless girl came to the door for a crust of bread, he said " No." If money was asked to build a church, he said " No." If he was solicited to make a contribution to aid in building a comfortable home for orphan children, he said "No." When his early school-mate was burned out and wanted an "extension" until he could "make collections," he said "No."

And thus it was he went through life. He never lent a helping hand to any human being; his heart was closed to all human sympathies; he had only one love, and that was for "gold;" his meat, his drink, his sunshine, the absorbing idol of his heart, was to get gold; and in order to keep it, he had the ever ready, the talismanic "No" to prevent him from risking a dime, or giving a penny to cheer any heart, to alleviate any sorrow, to counterbalance any calamity. It requires no stretch of the imagination to picture the old age and the dying hour of such a disgrace of his kind; loving no one, loved by no one, without any ennobling emotion, without any human sympathies, without love, without heart, without anything but hoarded gold. This was the man, the watchword of whose life was "No;" and when that life went out in an unlamented death, there was no tear shed at his grave, and no human being ever planted a flower near his tomb.

But there was another man, who quite as early in life learned to say "Yes." He was never known to refuse to do a real kindness. He was never known to reject an appeal for help. With all this, he had a rare sagacity to discriminate between the help asked and the help which would do the most good. But help he would. Said a poor, shiftless, idle vagabond one day to him, "Won't you please help me to a loaf of bread? I have had nothing to eat since yesterday morning, and it is so cold, and I am so hungry."

"Help you to a loaf of bread! why, certainly I will! just tumble this load of coal into the scuttle-hole, and I'll give you two loaves."

"But I have no shovel."

"Why, so you haven't, I might have known that; but lend a hand anyhow; here is the kitchen shovel, and I'll show you how to handle it."

But one day an old neighbor of his came to him in great distress. He had lost a cargo of great value by shipwreck; and unless he could raise five thousand dollars that day, he must suspend business, and lose the labor of twenty years.

" Lend you five thousand dollars! why, bless your soul, my old boy, I would just as lieve lend you twenty. Didn't we use to room together at college? haven't I always kept my eye on you ever since? and don't I know that you never let a note go to protest, that you have always been ready to discount your own unmatured paper, and that once, when your bank failed and " locked up " a large deposit, you paid a thousand dollars extra interest, rather than fail to meet a note on the day it was due, even when the holder had lost the right of protest?"

That man grew more kindly every day. He had a pleasant word for everybody, and a smile of recognition for every person. As years rolled on, his money increased without any effort of his own; his heart expanded wider and wider towards the strugglers around him; and when the coffin was uncovered before the pulpit, to allow the last look of friendship and affection, it took a whole hour for the crowd to pass by; and few there were who did not drop a real tear. The motto of this man's life was to say " Yes " to every appeal for " help." To those who deserved it he gave direct help, prompt, cheerful, and whole-hearted; the thriftless he helped to help themselves in a way to cultivate self-reliance without placing themselves under demoralizing obligations. JOSEPH JOHN GURNEY was the representative man of this class. After this will the reader learn to say " No " or " Yes?" To decide this, bear one thought in mind, that the moment you come to the conclusion that you will never help a human being as long as you live, that moment you have become a human brute, and had better be dead than alive, and not a living creature in the universe will " miss " you.

Still another lesson, beware of scrap philosophy. Have no inflexible rule but that of mercy and of right, and " thy days shall be long in the land which the Lord thy God giveth thee."

